# Upregulated FGFR1 expression is associated with the transition of hormone-naive to castrate-resistant prostate cancer

**DOI:** 10.1038/bjc.2011.367

**Published:** 2011-09-27

**Authors:** K Armstrong, I Ahmad, G Kalna, S S Tan, J Edwards, C N Robson, H Y Leung

**Affiliations:** 1 Solid Tumour Target Discovery Group, Northern Institute for Cancer Research, Newcastle University, Newcastle NE2 4HH, UK; 2 Urology Research, Beatson Institute for Cancer Research, Garscube Estate, Switchback Road, Glasgow G61 1BD, UK; 3 Experimental Therapeutics, Institute for Cancer Studies, University of Glasgow, Glasgow G12 8QQ, UK

**Keywords:** prostate cancer, formalin-fixed, paraffin-embedded samples, gene expression analysis, hormone-naive and castrate-resistant prostate cancer, fibroblast growth factor receptor 1

## Abstract

**Background:**

Prostate cancer (PC) represents a global health issue. Treatment for locally advanced and metastatic PC remains unsatisfactory. The androgen receptor (AR) has been validated in having a key role in both naïve and castrate-resistant PC (CRPC). However, the significance of other signalling pathways in CRPC is less well validated.

**Methods:**

To gain a better insight into the molecular signalling cascades involved in clinical CRPC, we performed gene expression profiling using the Illumina DASL assay and studied matched hormone-naive (HN) and CR prostate tumours (*n*=10 pairs). Ingenuity Pathways Analysis (IPA) was used to identify potential networks involved, and further validation was performed in *in vitro* cell models and clinical tumours.

**Results:**

Expression of 50 genes was significantly different between HN and CRPC. IPA revealed two networks of particular interest, including AR and FGFR1, respectively. FGFR1 expression was confirmed to be significantly upregulated in CRPC (*P*⩽0.005), and abnormal FGFR1 expression was associated with shorter time to biochemical relapse in HNPC (*P*=0.006) and less favourable disease-specific survival in CRPC (*P*=0.018).

**Conclusion:**

For the first time, our gene expression profiling experiment on archival tumour materials has identified upregulated FGFR1 expression to be associated with PC progression to the CR state.

Prostate cancer (PC) is the most commonly diagnosed cancer in men in the developed world, and its incidence continues to rise in most countries (Ferlay *et al*, 2007). There is a major unmet need to improve treatment for patients who develop castrate-resistant PC (CRPC), which remains incurable (Scher *et al*, 2005). Recent studies have confirmed involvement of persistent androgen receptor (AR) signalling in CRPC (Watson *et al*, 2010). The significance of this is further supported by the demonstrated clinical benefit with androgen ablation achieved by abiraterone treatment (Reid *et al*, 2010). However, how signalling cascades interact and modulate AR to contribute to CRPC remains poorly understood. In addition, non-AR-mediated signalling in CRPC has been predominantly studied in pre-clinical models, using *in vitro* cell studies and *in vivo* animal models. These studies have proposed the involvement of multiple signalling cascades and tumour suppressor genes in the pathogenesis of CRPC (Aggarwal and Ryan, 2011). However, the clinical relevance of many of these pathways remains to be validated. Archival formalin-fixed, paraffin-embedded (FFPE) tissue and associated clinical data provide a valuable resource to link gene expression data with tumour biology and patient outcome. We applied the cDNA-mediated annealing, selection, extension and ligation (DASL) assay (Illumina, San Diego, CA, USA; Bibikova *et al*, 2008) in paired hormone-naive (HN) and CR prostate tumour samples. Our objective was to identify key pathways implicated in the evolution of CR disease.

## Materials and methods

### Cell lines and reagents

LNCaP cells were obtained from the American Type Culture Collection (Manassas, VA, USA) and maintained in RPMI 1640 media supplemented with 10% fetal calf serum (Sigma, St Louis, MO, USA) at 37 °C in 5% CO_2_ atmosphere. CdxR-LNCaP cells were generated and maintained as described previously (Halkidou *et al*, 2003; Rigas *et al*, 2007). Anti-FGFR1 antibody (Flg (C-15)) was obtained from Santa Cruz Biotechnologies (Santa Cruz, CA, USA).

### RNA isolation and clinical samples

RNA was isolated from cell lines using TRIzol reagent (Invitrogen, Carlsbad, CA, USA) then treated with DNA*free* (Ambion, Austin, TX, USA) to remove any contaminating DNA according to the manufacturer's protocol.

Gene expression analysis was performed on matched HN and LHRH-relapsed (or CR) samples obtained by transurethral resection of the prostate (TURP) from 10 patients. Biochemical relapse signifying CRPC was defined both biochemically and histologically. The cohort of patients for HN and CR cancer were selected for analysis if they initially responded to hormone treatment (response was defined by prostate-specific antigen (PSA) levels decreasing by at least 50%), but subsequently relapsed (two consecutive increases in PSA of >10%) and had a pre-hormone and post-hormone relapse sample available for analysis. All patients received hormone therapy in the form of androgen deprivation therapy (either subcapsular bilateral orchidectomy and GnRH analogue) or maximum androgen blockade. To meet the inclusion criteria for matched-HN and -CR tumours, a response to this therapy had to be observed; a response to this therapy was defined by the PSA levels decreasing by at least 50%, and a nadir being reached. The hormone-sensitive tumour samples were obtained either from a TURP or prostatic needle biopsies. In addition, patients were required to relapse with CRPC to meet the inclusion criteria. A tumour was classified as CR if the patient stopped responding to hormone therapy, which was signified by two consecutive increases in their PSA levels. CR tumour samples were obtained by TURP, and all patients in this cohort initially respond to hormone therapy, but subsequently relapsed with hormone-refractory disease. All clinical samples were used in accordance with approval granted by the local hospital ethics committee.

FFPE tissue specimens were analysed histologically to identify tumour-rich regions, and samples (*n*=3) were taken using a 1-mm diameter core punch with the MTA-01 personal tissue arrayer (Beecher Instruments, Sun Prairie, WI, USA). Cores were deparaffinised in xylene (2 × 10 min), re-hydrated in 100, 90, 70 and 50% ethanol (5 min each) then air-dried. Tissues were digested in 100 *μ*l Proteinase K digestion buffer with 15 *μ*l Proteinase K (60 U *μ*l^−1^) (Ambion) at 55 °C overnight to remove cross-links. RNA was isolated using the HighPure RNA paraffin kit (Roche, Branchburg, NJ, USA), which includes a DNase I step to remove contaminating DNA.

### DASL assay and the IPA

Generation of cDNA, DASL assay, hybridisation to the bead array and data collection were carried out as described previously (Fan *et al*, 2004; Bibikova *et al*, 2004a, 2004b). Results were analysed using the BeadStudio software (Illumina), with data normalised using the Cubic Spline method as described by Bibikova *et al*, (2004a, 2004b).

All detected genes were individually analysed. Genes with detection scores >0.99 were included in the analysis (detection score=1−*P*-value, determined whether the target sequence signal was distinguishable from the negative control). Genes were then determined as significantly altered if their DiffScore exceeded 13, which signifies *P*<0.05. The DiffScore statistic assesses the probability of differential expression between a reference sample and a condition on an array. Within this model, technical and biological errors are taken into account. Technical error was determined using a least squares fit of s.e. and mean for the beads on both the reference and condition arrays. Average intensity of negative controls on reference and condition arrays provided an estimate of biological variation. Furthermore, a *P*-value was determined by dividing the absolute difference between the expression reference and condition arrays by the sum of the technical and biological variation on both arrays. Therefore, DiffScore=10 × Sign(cond_mean_−ref_mean_)log_10 (p) (Chudin *et al*, 2006).

The ‘Core Analysis’ from IPA (Ingenuity System Inc., Redwood City, CA, USA; http://www.ingenuity.com/) was used to interpret the set of 50 genes with DiffScore >13 (*P*<0.05), either up- or downregulated between HN and CRPC. IPA identified a set of networks involving subgroups of genes from the 50 identified genes.

### Primer design and qRT-PCR

Reverse transcription was performed on 2-*μ*g RNA extracted from cell lines. Briefly, 0.4 mM dNTP mix (Sigma), 0.25 *μ*g Oligo d(T)_15_ (R&D Systems, Minneapolis, MN, USA) and 100 units M-MLV reverse transcriptase (Promega, Madison, WI, USA) was used in a 20-*μ*l reaction. Reactions were performed at 37 °C for 1 h followed by enzyme inactivation at 95 °C for 5 min, immediately followed by incubation on ice.

Primers were designed using Primer Express (Applied Biosystems, Carlsbad, CA, USA), restricting amplicons to intron/exon boundaries and products approximately 50 bp in length. Reaction efficiencies were between 90–110% (*R*^2^>0.98) and all primers displayed one peak upon melting curve analysis. Primers against FGFR1 were designed to detect all the nine alternative transcripts. All primers used were provided by VHBio (Gateshead, UK). qRT-PCR was carried out using SYBR Green Jumpstart Taq Readymix (Sigma) in 384-well clear optical reaction plates, using the ABI 7900HT real-time PCR system (Applied Biosystems) according to the manufacturer's protocol. Primers were used at a final concentration of 1 ng *μ*l^−1^. All cell line samples were tested in triplicate at least twice. Expression was normalised against HPRT1 expression and repeated twice. Data generated was analysed by absolute quantification using SDS v2.2 software (Applied Biosystems). Primer sequences are as follows: *RPL13a* (5′-GTA CGC TGT GAA GGC ATC AA-3; 5′-GTT GGT GTT CAT CCG CTT G-3), *FGFR1* (5′-ACA CCA AAC CAA ACC GTA TG-3′ 5′-TGT CCA ATA TGG AGC TAC GGG-3′), and *HPRT1* (5′-TTG CTT TCC TTG GTC AGG CA-3′ 5′-AGC TTG CGA CCT TGA CCA TCT-3′).

### Immunohistochemistry (IHC)

IHC was performed on FFPE sections using Flg (C-15) (Santa Cruz Biotechnologies), an antibody which has been fully validated (Berger *et al*, 1999). A PC tissue microarray (TMA, ethical committee approval, MREC 01/0/36) was used, comprising 223 patients: 164 primary PC, 23 benign prostatic hypertrophic (BPH) and 36 matched pairs of HN and CR tumour samples.

For the HN- and CR-matched TMA, the median age of the patients at diagnosis was 70, ranging from 49 to 81, and the median PSA at diagnosis was 32 ng ml^−1^ (range 3–126) with a median Gleason sum score for the HN tumours at 7 (ranging from 4 to 10). At diagnosis, 12 patients had metastatic disease; however, this increased to 28 at relapse. The median time to relapse was 2.66 years (inter quartile range 1.76–4.71). Follow-up data were available for 33 patients. The median time to death from relapse was 1.87 years (interquartile range 1.05–2.93), whereas the median time to death from diagnosis was 5.82 years (interquartile range 3.44–6.83). During the follow-up period, by definition, all patients in this cohort relapsed with CR disease; 25 died of their disease and 8 deaths were attributed to intercurrent disease. An incidence TMA of PC was used to compare FGFR1 expression between PC and BPH controls. The median age of patients at diagnosis was 71.2 years (minimum of 48.3 years, maximum of 92.4 years). Median Gleason score of the prostate tumours was 7 (minimum of 2, maximum of 9).

Expression of FGFR1 in each core was assessed using the weighted histoscore method (Edwards *et al*, 2003), grading staining intensity as negative (0), weak (1), moderate (2) and strong (3), before multiplying by the percentage of tumour cells within each category. The final histoscores ranged from a minimum of 0 to a maximum of 300. Quantification of high/low was defined as above or below the median, respectively. This was graded separately by two observers (SST and IA), blinded to all outcome data. Inter-observer agreement was excellent with interclass correlation scores >0.80.

### Statistical analysis

Statistical evaluation was performed using the Mann–Whitney test to compare differences in FGFR1 expression between cancer and benign control samples. Paired-sample analysis was used to compare changes in protein expression between HN and CR samples. As individual pairs of these samples were from the same patient and were therefore related, it was considered that the Wilcoxon signed-rank test was an appropriate analysis to be used. Disease-specific survival rates were generated using the Kaplan–Meier method. The log-rank test was used to compare significant differences between the staining intensities. The Pearson correlation coefficient was used to test for correlation. Analyses were performed using the Statistical Package for Social Sciences software (Version 17.0; SPSS, Chicago, IL, USA).

## Results

### Gene expression analysis to compare clinical HN- and CRPC

**Figure 1:**
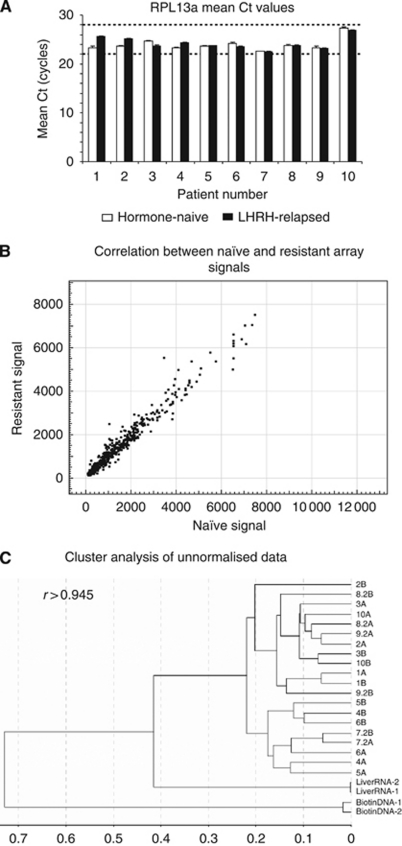
Pre-qualification of samples and evaluation of technical array data. (**A**) RPL13a expression in 10 paired hormone-naive and LHRH-relapsed samples. RPL13a expression was determined by qRT-PCR. Data is expressed as mean Ct values±s.d. qRT-PCR was performed in triplicate (*n*=2). (**B**) Naïve and resistant sample groups show a linear relationship. (**C**) Cluster analysis of unnormalised data reveals that all samples are highly correlated.

Ten matching pairs of HN and CRPC samples were studied. RNA isolated using the HighPure RNA Paraffin Kit (Roche) was quantified using RiboGreen (Invitrogen) with concentrations ranging between 66–324 ng *μ*l^−1^, satisfying the required minimal concentration (50 ng *μ*l^−1^) for DASL assay. In addition to RNA assay by the Agilent Bioanalyser (Agilent Technologies, Santa Clara, CA, USA), qRT-PCR was performed on all samples for ribosomal protein L13a (*RPL13a*) to generate a 90-bp fragment. The observed Ct values of 22 to 28 cycles excluded excessive degradation (Figure 1A) as determined by Bibikova *et al*, (2008). All samples were comparable in terms of their degradation status and no alterations in array quality were observed between samples. Analysis of matched-HN and -CRPC samples were performed over two arrays (Human Cancer Panel v1 containing 502 genes; Illumina), where matched pairs were applied to the same array, along with the relevant technical replicates and hybridisation controls. A linear relationship (*R*^2^=0.998; Figure 1B) was observed between the two data sets, signifying data comparability. Overall, 456 genes, out of 502 cancer-related genes within the cancer panel, were detected on both arrays with a detection score >0.99 (as defined in Materials and Methods). Cluster analysis of the data without normalisation confirmed that all samples were highly correlated with *R*>0.945 (Figure 1C).

**Figure 2:**
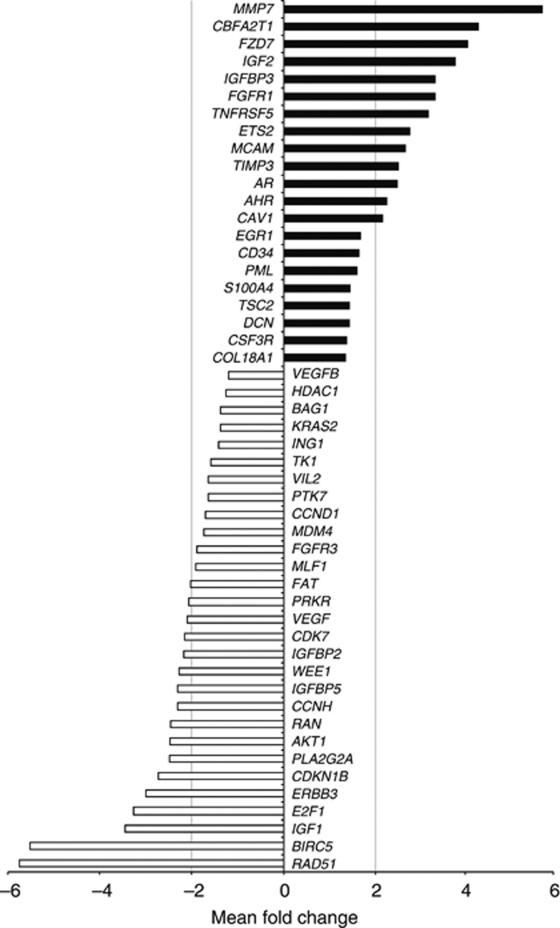
Differentially expressed genes between hormone-naive (HN) and castrate-resistant (CR) tumours. Fifty genes were determined as differentially expressed (DiffScore >13). Solid and open bars represent genes upregulated or downregulated, respectively, during transition from HN to CR status. The mean fold change in gene expression is represented in the *x* axis.

Among the 456-detected genes, the expression of 50 genes were significantly different between HN and CR tumours (DiffScore >13; *P*<0.05) as determined using BeadStudio software, with 21 genes upregulated and 29 genes downregulated (Figure 2). Thirteen genes were upregulated in CR tumours by more than two-fold, including *AR*, *CAV1*, *ETS2*, *FGFR1*, *IGF2, IGF-BP3, MMP7, TIMP3* and *TNFRSF5*, whereas 16 genes were downregulated by more than two-fold, including *AKT1, CDK7, ERBB3, IGF1, IGF-BP5*, and *VEGF*. Interestingly, molecules within the same signalling pathway can be differentially regulated during the HN to CR transition, including *FGFR1* (↑) and *FGFR3* (↓), *IGF2/IGF-BP3* (↑) and *IGF1/IGF-BP5* (↓), highlighting potential complex relationship between members of individual signalling pathways in CR prostate carcinogenesis.

**Figure 3:**
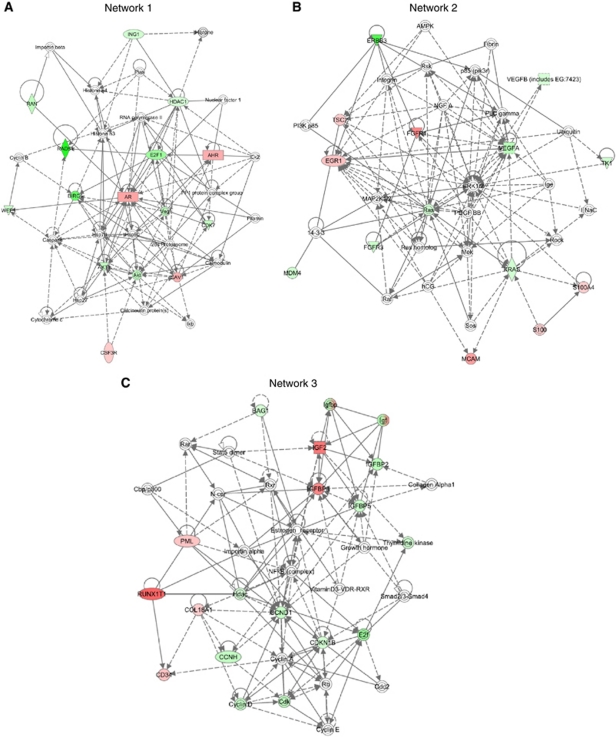
Network 1 (**A**), 2 (**B**) and 3 (**C**) were identified by the core analysis using IPA software, to be associated with the HN to CR transition. Details of symbols can be found at http://www.ingenuity.com/. In brief, red and green colours represent input molecules that are upregulated or downregulated, respectively. Additional molecules from the Ingenuity Knowledge Base, shown in white colour, connect the input molecules into a larger meaningful network.

To carry out formal assessment of the global signalling abnormalities associated with the development of CR disease, the ‘core analysis’ from IPA software (http://www.ingenuity.com/) was performed on the 50 differentially expressed genes between HN and CR tumours. IPA identified six heuristic networks that contain 13, 12, 12, 7, 5 and 2 genes from the list of 50-identified genes, respectively. The top ranked network involved 13 of the 50 genes (network 1; Figure 3A; Supplementary Table) including *AR*; the top functions associated with this network are tissue development, cell cycle and gene expression. The second ranked network implicated in the HN to CR transition identified 12 of the 50 genes, six of which were upregulated (Figure 3B; network 2 in Supplementary Table); the top functions of network 2 are tumour morphology, developmental disorder and genetic disorder. A third network, referred to as network 3, which is highly enriched for nuclear receptor network components, and identified 12 genes, but only three of these genes were upregulated, namely *IGF-BP3, IGF2* and *Runx1T* (Figure 3C; Supplementary Table). We are particularly interested to identify novel oncogenic activation associated with the transition from HN to CRPC. Hence, networks 1 and 2 are particularly interesting to our project.

Within network 1, AR appears to be critically involved as a signalling hub interacting with the majority of implicated genes in this network. In addition, *AR* has been extensively investigated and validated in both HN and CR disease (Shen and Balk, 2009). Hence, our focus turned to network 2. Twelve genes were implicated in network 2; the upregulated genes were *EGR1, FGFR1, MCAM, TSC2, S100* and *S100A4*, whereas *FGFR3, VEGFa/b, ras, MDM4* and *TK1* were downregulated. Within the FGFR system both *FGFR1* and *FGFR3* are highlighted in this network. Significantly, the degree of upregulation for *FGFR1* at ∼three-fold was highest within network 2. As expected, FGFR1 signalling closely links to ERK1/2. FGFR1 can also crosstalk with other receptor tyrosine kinases, including FGFR3 and erbB3. It is worth noting that the expression of *VEGFa/b* was reduced in CRPC. FGF- and VEGF-mediated signalling critically controls angiogenesis during tumourigenesis. VEGFR and FGFR may co-operate to promote neo-angiogenesis. Therefore, upregulated *FGFR1* expression may compensate for the reduced expression of *VEGFa/b* observed in network 2. Work from our group and others have previously implicated abnormal FGFR1 expression in prostate carcinogenesis (Devilard *et al*, 2006; Acevedo *et al*, 2007; Sahadevan *et al*, 2007). However, the role of FGFR1 in the development of CRPC has not been previously described. Overall, network 2 appeared to be novel, and we wish to validate the potential role of FGFR1 in both HN and CRPC, as its upregulation is ranked highest in this network. Our data therefore suggest that *FGFR1* may also have a role in CRPC. We further evaluated whether *FGFR1* expression is upregulated during the transition from HN to CR state using an *in vitro* cell model and clinical CRPC materials.

### FGFR1 in *in vitro* and clinical CRPC

**Figure 4:**
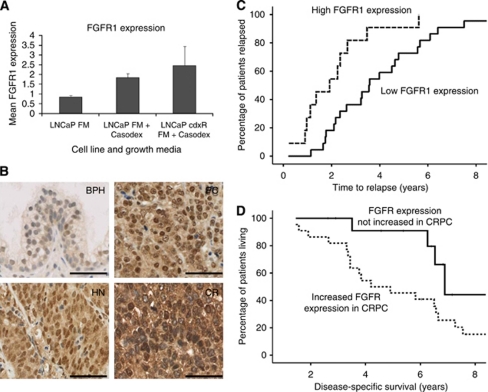
(**A**) qRT-PCR for FGFR1 expression in LNCaP and derived-casodex resistant (cdxR) cells. FGFR1 expression was normalised against HPRT1 expression and repeated twice. FM denotes full medium growth conditions, and casodex signifies the presence of bicalutamide (2 *μ*M). (**B**) Representative images for FGFR1 immunoreactivity in BPH, PC, and paired-HN and -CR tumours. Black bar represent 100 *μ*m. (**C**) Kaplan–Meier graph for the time to biochemical relapse in patients with HNPC as stratified according to FGFR1 expression. A total of 33 patients were included in the analysis, 22 classified as having low FGFR1 expression (in reference to the median value). By definition, all patients in this cohort had relapsed; therefore, there were also 22 events, as an event was defined by the patients experiencing relapse. In all, 11 patients were classified as having high FGFR1 expression, and similar to the above reasoning, there were also 11 events. (**D**) Kaplan–Meier graph for disease-specific survival in patients with CRPC as stratified according to whether FGFR1 expression was upregulated or not in CR disease, when compared with the respective HN tumours. Follow-up data on patient survival were available for 35 patients and were included in the analysis. In all, 22 patients were classified as having an increase in FGFR1 expression with development of CRPC with 20 events and 13 patients were classified as having no change in expression with 5 events. In this case, an event was a cancer-specific death.

Parental LNCaP cells were cultured continuously in the presence of 2 *μ*M bicalutamide (or casodex) for over 9 months. This resulted in the bicalutamide- (or casodex)-resistant phenotype; the bicalutamide-resistant LNCaP (referred to as cdxR-LNCaP thereafter) cells can be considered a model of CRPC (Rigas *et al*, 2007). In these cdxR-LNCaP cells with chronic AR antagonist treatment, FGFR1 expression was upregulated by about 2.5-fold (Figure 4A). Similarly, FGFR1 mRNA expression was upregulated by two-fold when AR function in LNCaP cells was acutely blocked by bicalutamide (2 *μ*M) for 48 h. These *in vitro* data is in keeping with the notion that abnormal FGFR1 expression is associated with CRPC, as well as HN disease.

Using IHC, prostate TMA containing 164 PC and 23 BPH samples were studied for FGFR1 expression (Figure 4B). We were able to validate our previous report of upregulated FGFR1 expression in PC, when compared with benign (BPH) control samples (*P*<0.0001; Sahadevan *et al*, 2007). To test the role of FGFR1 in CRPC, a TMA containing matched HN and CR tumours from 36 patients was studied. Histoscores of FGFR1 immunoreactivity were significantly increased during the HN to CR transition for both cytoplasmic (HN *vs* CR: mean 183 *vs* 212, median 200 *vs* 220, respectively, *P*=0.005) and nuclear (HN *vs* CR: mean 194 *vs* 234, median 200 *vs* 240, *P*=0.002) staining. Membranous FGFR1 staining was not observed in this study, and therefore not included in the analysis. Consistent with previous reports, expression of AR and Ki67 were significantly increased in CRPC when compared with HNPC (*P*<0.0001; Tan *et al*, 2010).

To determine whether FGFR1 overexpression is associated with progression of HN tumours to the CR state as defined by the time to biochemical relapse (persistent and significant rise in serum PSA levels), Kaplan–Meier graphs for tumours expressing low (below median) and high (above median) levels of FGFR1 were plotted and compared using the log-rank test. Patients with low FGFR1-expressing HNPC had a mean time to relapse of 3.9 years, whereas patients with tumours showing high cytoplasmic FGFR immunoreactivity relapsed quicker with a mean time to relapse of 2.1 years (*P*=0.006; Figure 4C). Nuclear FGFR1 expression was not associated with disease progression.

Kaplan–Meier graphs of tumours were plotted to analyse if enhanced FGFR1 expression in CRPC, when compared with matched HNPC, was related to patient survival outcome. We observed that an increase in (nuclear) FGFR1 expression from HN to CRPC was associated with reduced mean disease-specific survival of 4.9 years, compared with 7.4 years in those that did not show such enhanced FGFR expression in CR tumours (*P*=0.018; Figure 4D). Interestingly, we observed no difference with cytoplasmic expression of FGFR1.

Taken together, upregulated FGFR1 expression is important in prostate carcinogenesis, and we report for the first time its involvement in both HN and CRPC.

## Discussion

Treatment for CRPC remains unsatisfactory, and novel-targeted therapy is urgently needed. Despite ongoing developments in taxane and androgen ablation therapies, including cabazitaxel and abiraterone, respectively, patients with CRPC continue to die prematurely. We studied matched HN and CR prostate tumours, using a focused Human Cancer Panel DASL analysis to identify genes involved in the HN to CR transition. Triplicate cores from individual tumour-rich areas were obtained from FFPE samples, and extracted RNA was confirmed to be adequate for analysis, with Ct values for the house-keeping gene, *RPL13a*, between 22 and 28 cycles (Bibikova *et al*, 2004a). Next-generation sequencing methodologies, along with the relevant amplification protocols are rapidly evolving. Our data highlight the feasibility of studies utilising laser-captured microdissected FFPE materials for genome, exome or RNA sequencing.

A total of 50 out of 502 genes in the cancer panel were significantly different between HN and CR tumours. Overrepresented signalling pathways were identified using the ‘core analysis’ of the IPA software. The top-ranked network identified AR to be a key component, in keeping with available data on continued AR contribution in CRPC (Edwards *et al*, 2003; Watson *et al*, 2010). The second-ranked network implicated FGFR1 to be involved in CRPC. Extending our previous work and reports from other laboratories (Devilard *et al*, 2006; Sahadevan *et al*, 2007), we further validated the association of FGFR1 overexpression with CRPC and its impact on clinical outcome: (1) increased risk of developing CRPC with shorter time to relapse when FGFR1 expression is elevated in HN tumours, (2) higher levels in CR tumours when compared with HNPC, and (3) shorter time to death for CRPC with elevated FGFR expression.

The association of nuclear FGFR1 overexpression with reduced patient survival is interesting and warrants further investigation in a larger cohort of patients with CRPC for more robust subgroup survival analysis. It is worth noting that in HNPC, cytoplasmic FGFR1 was associated with disease progression to CRPC, whereas enhanced nuclear FGFR1 expression was found to be related to shorter patient survival in established CR disease. Given the relatively small sample size of this paired TMA, future studies are required to study the significance of nuclear and cytoplasmic FGFR1. Nuclear FGFR1 cooperates to regulate FGF-2, ribosomal S6 kinase (RSK1) and CREB-binding protein, with RSK1 capable of further promoting nuclear localisation of FGFR1 (Dunham-Ems *et al*, 2006, 2009).

We applied bicalutamide as an *in vitro* model for androgen ablation therapy. Both acute and chronic bicalutamide-treated LNCaP cells showed significantly upregulated FGFR1 expression (>two-fold). Interestingly, when cultured in charcoal-striped medium, LNCaP cells completely lost their FGFR1 expression. The underlying mechanism is unclear, but is likely due to the depletion of factors in addition to androgens. Given the close correlation for FGFR1 expression in our *in vitro* cell model and clinical tumour samples, as well as the specific mode of action for casodex (bicalutmide), we believe that the use of bicalutamide treatment is a valid *in vitro* tool for modelling androgen ablation.

Among the six networks identified by the core analysis using the IPA software, the top network (with 13 genes) confirmed previous data on the role of AR in CRPC. From network 2, we validated FGFR1 (with the highest fold of upregulation in this network) to be important in CR disease *in vitro* and in clinical tumours. The relationship between FGFR1 and the other implicated signalling molecules in network 2 warrants further mechanistic analysis, for which the cdxR-LNCaP cells is a useful model. Interestingly, a number of genes identified in network 2 have been implicated in drug resistance in cancers (Mencia *et al*, 2010; Parra and Ferreira, 2010). Indeed, FGFR1 itself has been linked with tamoxifen resistance in breast cancer (Turner *et al*, 2010) and cisplatin resistance in ovarian cancer (Cole *et al*, 2010). Amplification of FGFR1 has been reported in breast cancers (Turner *et al*, 2010) and relapsed-PC (Edwards *et al*, 2003), which may provide a mechanism for the upregulation seen here. Yet, there is still evidence that upregulation of FGFR1 transcription may have a role, based on cell line findings where casodex was applied for 48 h (Figure 4A). Together, this would result in cells being more sensitive to FGFR1 ligands and potential ligand-independent activation. However, the molecular basis in which FGFR1 contributes to CRPC requires further analysis to address whether FGFR1 serves a distinct role in HN and CR disease. Although network 3 identified 12 genes from the 50 candidate genes, only 3 were upregulated. Nonetheless, it will be important for future studies to explore the impact of these genes (both up- and downregulated) in CRPC.

Prostate-specific activation of FGFR1 in the epithelial compartment results in epithelial-to-mesenchymal transition and development of adenocarcinoma in 100% of cases. Interestingly, deactivation of FGFR1 in early cancers leads to complete tumour regression, suggesting a role in both initiation and progression (Acevedo *et al*, 2007), As we have discovered significant association between FGFR1 upregulation and CRPC, future investigation using castration experiments on this transgenic FGFR1 PC mouse model may provide insight into the impact of abnormal FGFR1 function in the development of CRPC. It is also worth noting that despite the earlier observation of *in vitro* activation of the AR by growth factors, including members of the FGF family (Culig *et al*, 1994), whether aberrant FGFR signalling may contribute to prostate carcinogenesis via ligand-independent activation of AR remains unclear. To our knowledge, somatic mutations of *FGFR1* are not involved in human PC. Interestingly, miR-16 is thought to regulate several target genes including *FGFR1* (Gatt *et al*, 2010) and may be responsible for aberrant FGFR1 expression in PC. Future investigations can assess if miR-16 expression is progressively lost with the transition from HN to CR disease. The three-fold induction of *FGFR1* expression observed in the development of CRPC is the highest among the genes activated in the second-ranked network, and along with the reported contribution of *FGFR1* in PC biology, we would propose that *FGFR1* is the gene of interest rather than a proxy or downstream effect of an independent tumour-promoting event.

In summary, our gene expression analysis using a focused cancer-associated gene set supported previous data on AR and uncovered the association of *FGFR1* in CRPC.
