# Supplementary material for: Upregulated FGFR1 expression is associated with the transition of hormone-naive to castrate-resistant prostate cancer
Source: Br J Cancer. 2011 Sep 27;105(9):1362–9. doi: 10.1038/bjc.2011.367 (PMC3241546; doi:10.1038/bjc.2011.367)
Supplement: Supplementary Table 1 [file bjc2011367x1.doc]

**Supplementary Table.**

Network 1

| **Gene** | **Fold change** |
| --- | --- |
| **Androgen receptor (AR)** | **2.5** |
| caveolin 1 (CAV1) | 2.2 |
| aryl hydrocarbon receptor (AHR) | 2.2 |
| AKT1 | -1.9 |
| cyclin-dependent kinase 7 (CDK7) | -1.9 |
| RAN GTPase (RAN) | -1.9 |
| VEGFA | -2.1 |
| E2F transcription factor 1 (E2F) | -2.9 |
| baculoviral IAP repeat containing 5 (BIRC5) | -4.5 |
| RAD51 homolog (RAD51) | -6.7 |

Network 2

| **Gene** | **Fold change** |
| --- | --- |
| **FGFR1** | **3.3** |
| melanoma cell adhesion molecule (MCAM) | 2.7 |
| early growth response 1 (EGR1) | 1.7 |
| tuberous sclerosis 2 (TSC2) | 1.4 |
| S100 calcium binding protein A4 (S100A4) | 1.3 |
| thymidine kinase 1 (TK1) | -1.4 |
| MDM4 | -1.4 |
| FGFR3 | -1.6 |
| VEGF-A | -2.1 |
| ERBB3 | -5.3 |

Network 3

| **Gene** | **Fold change** |
| --- | --- |
| runt-related transcription factor 1 (RUNX1T1) | 4.3 |
| insulin-like growth factor 2 (IGF2) | 3.7 |
| IGFBP3 | 3.3 |
| insulin-like growth factor binding protein 5 (IGFBP5) | -1.6 |
| cyclin D1 | -1.7 |
| cyclin H | -1.8 |
| CDK7 | -1.9 |
| insulin-like growth factor binding protein 2 (IGFBP2) | -2.0 |
| IGF1 | -2.3 |
| E2F1 | -2.9 |
