# Supplementary material for: Upregulated FGFR1 expression is associated with the transition of hormone-naive to castrate-resistant prostate cancer
Source: Br J Cancer. 2011 Sep 27;105(9):1362–9. doi: 10.1038/bjc.2011.367 (PMC3241546; doi:10.1038/bjc.2011.367)
Supplement: Supplementary Table Legend [file bjc2011367x2.doc]

**Supplementary Table. Summary of top ten genes within Networks 1-3.** Genes are ranked according to the absolute fold increase or decrease in expression between HN and CR PC.
